# Clinical outcomes and impact of prognostic factors in resectable oral cavity squamous cell carcinoma

**DOI:** 10.3389/fonc.2024.1443367

**Published:** 2024-10-11

**Authors:** Shatha Abu Taha, Taher Abu Hejleh, Lina Wahbeh, Abdulla Alzibdeh, Mohammad Berawi, Mohamed Qambar, Mohammad Mukahal, Fawzi Abuhijla, Ramiz Abu-Hijlih, Ayat Taqash, Tariq Hussein, Medyan Alrousan, Omar Al Saraireh, Wisam Al-Gargaz, Akram Al-Ibraheem, Hamza Ghatasheh, Ali Hosni, Issa Mohamad

**Affiliations:** ^1^ Department of Radiation Oncology, King Hussein Cancer Center, Amman, Jordan; ^2^ Department of Medical Oncology, King Hussein Cancer Center, Amman, Jordan; ^3^ Department of Biostatistics, King Hussein Cancer Center, Amman, Jordan; ^4^ Department of Internal Medicine, Ibn Al-Hytham Hospital, Amman, Jordan; ^5^ Department of Surgical Oncology, King Hussein Cancer Center, Amman, Jordan; ^6^ Department of Special Surgery, Jordan, University of Science and Technology, Irbid, Jordan; ^7^ Department of Nuclear Medicine, King Hussein Cancer Center, Amman, Jordan; ^8^ Radiation Medicine Program, Princess Margaret Cancer Centre, University Health Network, University of Toronto, Toronto, ON, Canada

**Keywords:** oral cavity squamous cell carcinoma, surgery, adjuvant therapy, radiotherapy, chemoradiotherapy, clinical outcomes, prognostic factors

## Abstract

**Purpose:**

To evaluate clinical outcomes and prognostic factors in non-metastatic oral cavity squamous cell carcinoma (OCSCC) patients who underwent surgery with or without adjuvant therapy.

**Methods:**

From 2007 and 2018, 116 patients were analyzed. The primary endpoint was overall survival (OS), and secondary endpoints were disease-free survival (DFS), local failure (LF), regional failure (RF), and distant metastases (DM). Kaplan-Meier method and log-rank test assessed survival outcomes, while Cox proportional hazard tests analyzed prognostic factors.

**Results:**

Median patient age was 53 years, most were smokers (93.5%) and males (62.9%). Predominant subsite was the oral tongue (58.6%). Treatment included surgery alone (16.4%), adjuvant radiotherapy (46.6%), or adjuvant concurrent chemoradiotherapy (CCRT) (37%). The median follow-up time was 45.9 months. There were significant differences between groups in terms of gender (P=0.028) and RT dose (P=0.01). The 3-year OS, DFS, LF, RF and DM for the entire cohort were 60.9%, 55.1%, 20.11%, 8.43%, and 17.13%, respectively. Surgery alone yielded higher 3-year OS (81.4%) than adjuvant RT (70%) or adjuvant CCRT (41.4%), (p=0.012). Adjuvant CCRT correlated with higher LF compared to adjuvant RT and surgery alone groups (p=0.029). Lymphovascular invasion (LVI) impacted OS (HR=2.034, p=0.0498) and DM (HR=3.380, p=0.0132), while higher tumor grade increased DM likelihood (HR=8.477, p=0.0379).

**Conclusions:**

This study reports OCSCC patient outcomes in Jordan across different treatment modalities. Adjuvant CCRT correlated with higher LF rates, and LVI impacted OS and DM, aligning with existing OCSCC treatment literature.

## Introduction

Oral cavity cancer stands as one of the most common malignancies globally ranking among the top 10 incident cancers (1). The most common histology is squamous cell carcinoma (SCC). Tobacco and alcohol use are the main etiologic factors (2). Other risk factors include poor oral hygiene, betel nut chewing and immune suppression (3). Incidence of oral cavity squamous cell carcinoma (OCSCC) in the Arab countries is variable. In Syria, the incidence is 0.5/100,000 while it reaches up to 10/100,000 in the Southern parts of Saudi Arabia (4). In the Middle East and North Africa (MENA), newly diagnosed cases of OCSCC represents 1.5% of all malignancies (5). The crude incidence and mortality rates of these cancers are expected to double by 2030 (5). In Jordan, 97 new cases of OCSCC were reported in 2020, with 47 deaths due to the disease (6). The incidence is approximately twofold in males compared to females, possibly due to more frequent use of tobacco and alcohol among males (6).

The primary intervention for resectable OCSCC is surgery followed by risk-adapted postoperative radiotherapy (PORT) with or without concurrent chemotherapy (7, 8). Patients with higher risk of locoregional failure require PORT, such as those with pT3-4, pathologic nodal involvement (pN+), close resection margin, and other pathologic features e.g., high histologic grade, peri-neural invasion (PNI), lymphovascular space invasion (LVI), depth of invasion, and pENE (9). A combined analysis of the RTOG 9501 and EORTC 2291 phase III randomized controlled trials (10) showed that patients with pathologic extranodal extension (pENE) and/or involved resection margin(s) benefited from the addition of concurrent chemotherapy to postoperative RT.

This retrospective study aims to report the clinical outcomes associated with OCSCC and assess the potential prognostic factors in patients with non-metastatic OCSCC who underwent surgery with or without adjuvant RT or CCRT, within a single institution in Jordan.

## Materials and methods

### Study population

This is a retrospective review of OCSCC patients treated at King Hussein Cancer Center (KHCC) from January 2007 to October 2018. After institutional review board (IRB) approval (IRB No 22 KHCC 150), we included OCSCC patients above 18 years who underwent curative-intent surgical resection +/- postoperative RT. Cancer stage was reviewed according to the American Joint Committee on Cancer (AJCC8^th^ edition) (11). Patients who had prior radiation to the head and neck (HN) area, radiation outside KHCC, received radiation dose less than 50Gy, or had poor Eastern Cooperative Oncology Group (ECOG) performance status >2 were excluded.

### Diagnostic approach

The multidisciplinary pre-treatment assessment and staging process included a thorough medical history, comprehensive physical examination, imaging evaluation with HN MRI scans, as well as PET/CT and/or chest CT scans. Before initiating postoperative RT, specialized teams of dentistry, nutritionists, and speech pathologists conducted pre-RT evaluations for each patient.

### Treatment approach

Patients with OCSCC are treated primarily with upfront surgical resection of the primary tumor with neck dissection. This is followed by adjuvant RT if the patient was considered at-risk of locoregional recurrence (e.g., close resection margin[s, pT3-4, pN2–3, involved node at levels IV-V, or presence of combined risk features e.g., PNI, LVI, high histologic grade, and pN1). If the patient had positive resection margin(s) and/or pENE, then they were considered to receive adjuvant platinum based CCRT (if deemed clinically suitable).

RT, ideally started within six weeks of surgery, and was delivered using 3‐dimensional conformal radiotherapy (3D‐CRT, until 2012) or intensity‐modulated radiation therapy (IMRT, after 2012) techniques (12). RT was given at a dose of 60Gy in 30 fractions if standard-risk, 66Gy in 33 fractions if high risk without residual/recurrent gross disease, and 70Gy in 35 fractions if early locoregional recurrent gross disease (i.e., prior to the planned postoperative RT). For patients who received adjuvant CCRT, the concurrent radiosensitizing chemotherapy was high‐dose cisplatin (100 mg/m^2^ given on days 1, 22, and 43) or low‐dose cisplatin (40 mg/m^2^ weekly during RT). For patients with contraindication to cisplatin, weekly carboplatin (area under the curve (AUC =1.5) was used instead of cisplatin.

### Post-treatment evaluation and follow-up

Post-treatment imaging included HN MRI scans and PET/CT, conducted 10–12 weeks post-RT completion. Subsequent follow-up appointments occurred every three months during the initial two years, then every four months in the third year, every six months in the fourth and fifth years, and annually thereafter.

### Statistical methods

The primary endpoint was OS. Secondary endpoints were disease-free survival (DFS), local failure (LF), regional failure (RF), and distant metastases (DM). The OS and DFS were analyzed using the Kaplan-Meier method and compared using log-rank test. LF, RF, and DM rates were calculated using the cumulative incidence method, with death as a competing risk. Outcomes were calculated from the date of diagnosis to the first event. Multivariable analysis (MVA) using Cox proportional hazards regression was used to identify predictors of OS, DFS and DM. All reported *p* values were 2-sided, with a statistical significance level of *p* < 0.05. All analyses were performed using SAS version 9.4 (SAS Institute Inc., Cary, NC), and the figures were generated using GraphPad PRISM 7.

## Results

### Patient, tumor, and treatment characteristics

Patient, tumor, and treatment characteristics are summarized in **Table 1**. A total of 116 patients with OCSCC were included in our study, with a median age of 53 years. Median follow-up time for the whole cohort was 46 (1.2-144 months), 73 (62.9%) were male, 101 (93.5%) were smokers, while only 4 (3.9%) drank alcohol. The most common subsite of disease was the oral tongue 68 (58.6%), followed by the buccal mucosa 17 (14.7%).

**Table 1:** Demographic, clinical, and treatment characteristics of patients with oral cavity squamous cell carcinoma.

Variable n, (%)	Whole cohort	Subgroups			*p* value
	N = 116	Surgery N = 19 (16.4%)	Adjuvant chemoradiotherapy N = 43 (37%)	Adjuvant radiotherapy N = 54 (46.6%)	
**Follow-up**, median (range), (months)	45.9 (1.2- 144)	57.8(1.2-144)	42.5(4.6-128)	43.6(3.4-135)	0.757
**Age** median (range), (years)	53 (23- 91)	57 (34-91)	52(23-80)	54(23-80)	0.585
**Gender**					**0.028**
* Male*	73(62.9%)	7(36.8%)	31(72.1%)	35(64.8%)
* Female*	43(37.1%)	12(63.2%)	12(27.9%)	19(35.2%)
**ECOG PS**					0.856
* 0*	108(94.7%)	17(94.4%)	39(92.9%)	52(96.3%)
* 1*	6(5.3%)	1(5.6%)	3(7.1%)	2(3.7%)
*Not reported*	2(0%)	1(0%)	1(0%)		
**Smoking status**					0.444
* No*	7(6.5%)	2(11.8%)	3(7.3%)	2(4%)
* Yes*	101(93.5%)	15(88.2%)	38(92.7%)	48(96%)
* Not reported*	8(0%)	2(0%)	2(0%)	4(0%)	
**Alcohol Drinking status**					0.827
* No*	98(96.1%)	17(94.4%)	38(97.4%)	43(95.6%)
* Yes*	4(3.9%)	1(5.6%)	1(2.6%)	2(4.4%)
* No reported*	14(0%)	1(0%)	4(0%)	9(0%)	
**Subsite**					
* Lip*	0	0	0	0	NA
* Gingiva*	4(3.4%)	2 (10.5%)	0	2 (3.7%)	0.119
* Hard palate*	7(6%)	0	4(9.3%)	3(5.6%)	0.521
* Alveolar ridge*	7(6%)	1(5.3%)	1(2.3%)	5(9.3%)	0.329
* Buccal mucosa*	17(14.7%)	5(26.3%)	6(14%)	6 (11.1%)	0.269
* Retromolar trigone*	10(8.6%)	1(5.3%)	4(9.3%)	5 (9.3%)	0.999
* Tongue*	68(58.6%)	10 (52.6%)	24 (55.8%)	34 (63.0%)	0.657
* Floor of mouth*	11(9.5%)	0	5 (11.6%)	6 (11.1%)	0.366
**Neck dissection (N=104)**					0.301
* Bilateral*	19(16.4%)	1(5.3%)	5(11.6%)	13(24.1%)
* Unilateral*	85(73.3%)	16(84.2%)	34(79.1%)	35(64.8%)
* No*	12(10.3%)	2(10.5%)	4(9.3%)	6(11.1%)
**XRT dose, mean ± SD**	64.6 ± 3.79	NA	67.4 ± 2.4	62.4 ± 3.2	**0.01**
**XRT technique**					0.887
* 3DCRT*	21(21.6%)	0	9(21%)	12(22.2%)
* IMRT*	76(78.4%)	0	34(79%)	42(77.8%)

ECOG PS, Eastern Cooperative Oncology Group performance status; XRT, radiotherapy; 3DCRT, Three-dimensional conformal radiotherapy; IMRT; intensity modulated radiation therapy. Bold *p*-values denote statistical significance (P < 0.05).

Regarding treatment, 19 (16.4%) were treated with surgery alone, 54 (46.6%) received adjuvant RT, and 43 (37%) received adjuvant CCRT. 19 patients (16.4%) had bilateral neck dissection, 85 (73.3%) underwent unilateral neck dissection, while 12 patients (10.3%) managed with resection of the primary cancer without neck dissection. In those who received RT, the median dose was 66 Gy (range 58-74 Gy), and most patients were treated with IMRT (n=76,78.4%). There were significant differences between the treatment groups in terms of gender (p = 0.028), see **Table 1**. Pathological characteristics are summarized in **Table 2**.

**Table 2:** Pathologic characteristics of patients with oral cavity squamous cell carcinoma.

Variable n, (%)	Whole cohort	Subgroups			*p* value
	N = 116	Surgery N = 19 (16.4%)	Adjuvant chemoradiotherapy N = 43 (37%)	Adjuvant radiotherapy N = 54 (46.6%)	
**Tumor size** (cm), median (range)	2.5(0.6-7)	1.7(0.6-5)	3(0.6-7)	2.3(0.6-6)	**0.003**
**Depth of invasion** (mm), median (range) *N=58*	2.5(0.6-7)	6(1.6-16)	13.8(1.4-20)	10(0.5-22)	0.052
**Tumor thickness** (mm), median (range) *N=32*	21(0.0-25)	4.0(2-7.5)	22(1-25)	8.9(0-23)	**0.015**
**pT stage**					**0.01**
* T1-T2*	69(59.5%)	18(94.7%)	16(37.2%)	35(64.8%)
* T3-T4*	47(40.5%)	1(5.3%)	27(62.8%)	19(35.2%)
**pN stage**					**0.01**
* N0-N1*	67(57.8%)	18(94.7%)	16(37.2%)	33(61.1%)
* N2-N3*	49(42.2%)	1(5.3%)	27(62.8%)	21(38.9%)
**LN harvested**, mean ± SD	37.7 ± 23.4	30 ± 18.5	40.6 ± 21.5	38.2 ± 26.2	0.328
**LN positive**, mean ± SD	1.92 ± 4.22	0.4 ± 1.1	3.4 ± 6.3	1.1± 1.3	**0.01**
**Tumor grade**					0.095
* GX*	2(1.7%)	0	0	2(3.7%)
* G1*	30(25.9%)	9(47.4%)	8(18.6%)	13(24.1%)
* G2*	65(56%)	7(36.8%)	25(58.1%)	33(61.1%)
* G3*	19(16.4%)	3(15.8%)	10(23.3%)	6(11.1%)
**Margins**					**0.01**
* Close*	43(39.1%)	7(46.7%)	12(29.3%)	24(44.4%)
* Negative*	42(38.2%)	8(53.3%)	8(19.5%)	26(48.1%)
* Positive*	25(22.7%)	0	21(51.2%)	4(7.4%)
* Unknown*	6(0%)	4(0%)	2(0%)	0	
**LVI**					0.058
* Absent*	84(79.2%)	18(100%)	28(75.7%)	38(74.5%)
* Present*	22(20.8%)	0	9(24.3%)	13(25.5%)
* Unknown*	10(0%)	1(0%)	6(0%)	3(0%)	
**PNI**					**0.002**
* Absent*	65(63.1%)	17(100%)	19(50%)	29(60.4%)
* Present*	38(36.9%)	0	19(50%)	19(39.6%)
* Unknown*	13(0%)	2(0%)	5(0%)	6(0%)	
**ENE**					**0.01**
* Absent*	98(84.5%)	17(89.5%)	27(62.8%)	52(96.3%)
* Present*	18(15.5%)	0	16(37.2%)	2(3.7%)
* Unknown*	0	2(10.5%)	0	0	

pT, pathological tumor category; pN, pathological nodal category; LN, lymph node; G, grade; LVI, lymphovascular invasion; PNI, perineural invasion; ENE, extranodal extension. Bold *p*-values denote statistical significance (P < 0.05).

### Survival outcomes

**Figure 1:**
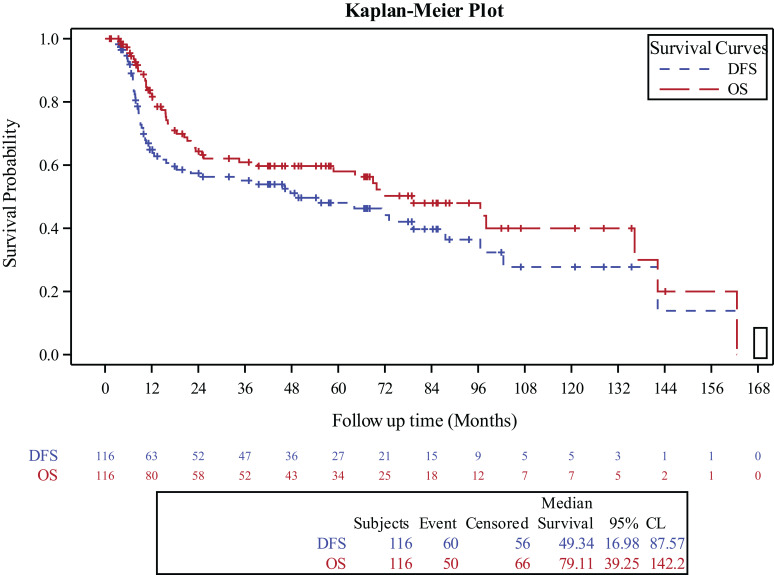
Kaplan-Meier curve for overall survival and disease free survival in the entire study population.

For the entire cohort, the 3‐year OS and DFS were 60.9% (95% CI, 50.9%-70.4%) and 55.1% (95% CI, 45.3%-64.7%), respectively (**Figure 1**). The 3-year OS for the surgery alone cohort was 81.4% (95% CI, 59.4%-96%), for the surgery followed by adjuvant RT cohort was 70% (95% CI, 55.5%-87.2%), and for the surgery followed by adjuvant CCRT cohort was 41.4% (95% CI, 26.2%-57.6%), (p=0.01). The 3-year DFS was 70.6% for the surgery alone cohort (95% CI, 47.5%-89.2%), 68.1% (95% CI, 54%,80.6%) for the surgery followed by adjuvant RT cohort (95% CI, 54%-80.6%), and 33.8% for the surgery followed by adjuvant CCRT cohort (95% CI, 19.9%-49.3%), (p=0.03).

### Tumor control outcomes

**Figure 2:**
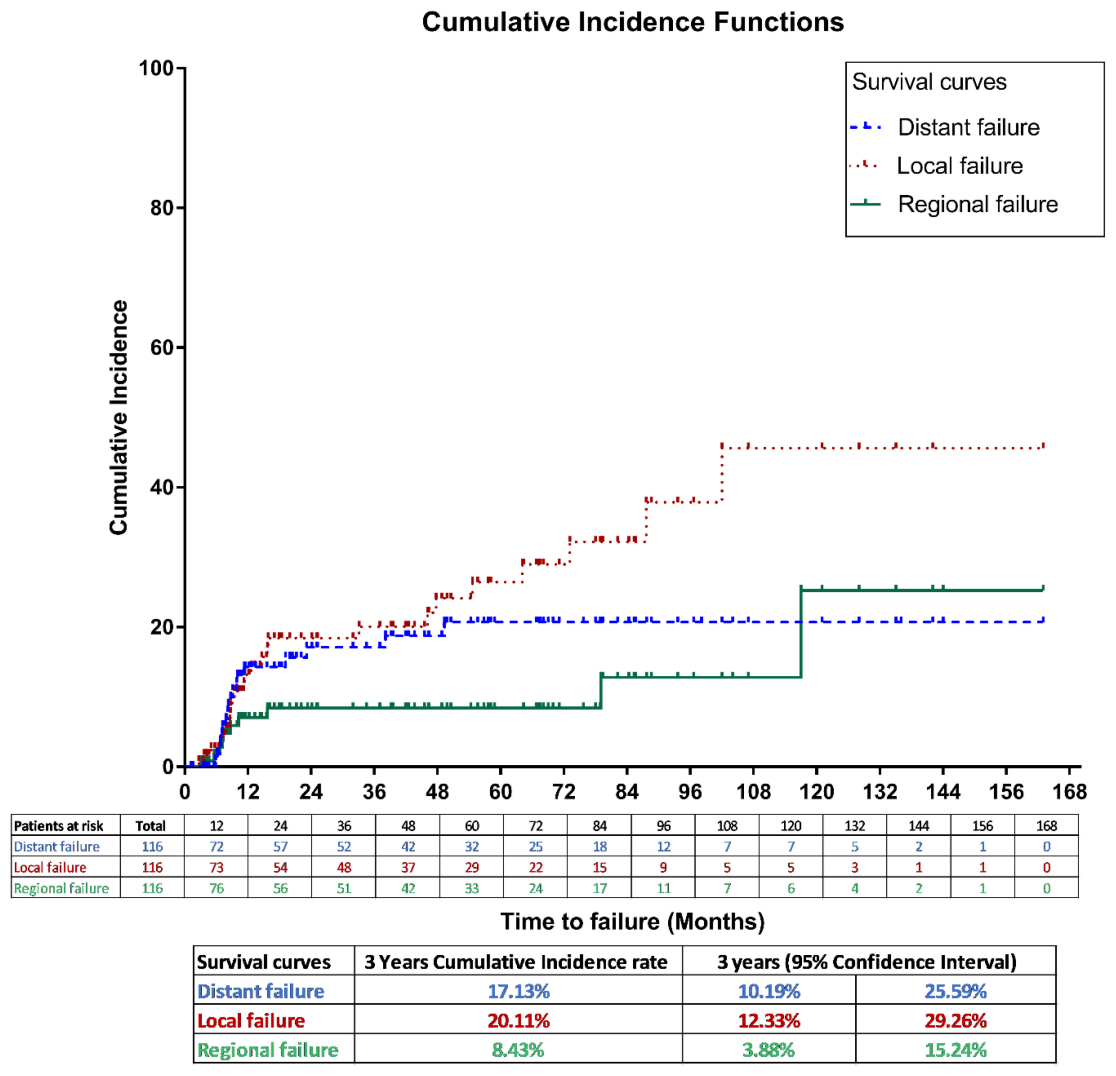
Cumulative incidence method for local, regional and distant failures in the entire study population.

For the entire cohort, the 3-year LF, RF and DM cumulative incidence rates were 20.11% (95% CI, 12.3%–29.4%), 8.43% (95% CI, 3.9% –15.2%), and 17.13% (95% CI, 10.2% – 25.6%), respectively, as shown in **Figure 2**.

Patterns of failure are shown in **Table 3**. For patients who underwent curative surgery alone, LF was observed in 6 cases after a median duration of 29 months post treatment (range 3-102).Among patients in this cohort who developed LF, half (n=3) were managed with salvage surgery followed by adjuvant RT, while the other half were managed with salvage surgery followed by adjuvant CCRT. RF occurred in 2 cases after a median duration of 10-and 117 months post-surgery, whilee DM were observed in 2 cases with a median duration of 18 and -38 months post-surgery. Among the two patients in this cohort who developed RF both had previously undergone unilateral neck dissection. One developed recurrence at bilateral level III cervical nodes, and was managed by salvage surgery followed by adjuvant CCRT, while the other developed recurrence at the ipsilateral previously dissected level II cervical nodes, and was managed by salvage surgery followed by adjuvant RT.

**Table 3:** Patterns of failure by type of treatment.

Cohort	Local failure (n= 25)			Regional failure (n=10)			Distant metastases (n=18)		
	n	Median (range), months	*p* value	n	Median (range), months	*p* value	n	Median (range), months	*p* value
**Curative surgery**	6	29.1 (3.2-102)	0.252	2	63.5 (10.2-117)	0.117	2	28.2 (18.4-38.1)	0.068
**Surgery followed by radiotherapy**	8	29.6 (5.0-72.5)	0.200	5	5.3 (1.7-78.4)	0.117	8	6.8 (5.0-48.2)	0.929
**Surgery followed by chemoradiotherapy**	11	7.9 (2.1-26.6)	**0.029**	3	7.9 (6.5-14.7)	0.732	8	6.9 (3.9-7.9)	0.214

Bold *p*-values denote statistical significance (P < 0.05).

In the cohort who received surgery followed by adjuvant RT, LF was reported in 8 cases with a median duration of 29.6 months post treatment (range 5.0-72.5), RF in 5 cases with a median duration of 5.3 months (range 1.7-78.4), and DM in 8 cases with a median duration of 6.8 months (range 5.0-48.2). Patients in this cohort who developed LF were managed with either salvage surgery followed by adjuvant re-RT (n=3), salvage surgery followed by adjuvant CCRT (n=2), re-RT (n=1), or CCRT (n=1). Among the patients in this cohort who developed RF, one had previously undergone unilateral neck dissection, and recurrence occurred in the contralateral undissected neck at level IV. Meanwhile, two had undergone bilateral neck dissection, developed recurrences at level II and III, and were treated with salvage surgery followed by adjuvant RT. Two others had had no neck dissection done in the past, developed recurrence at level II, and were managed in one instance by CCRT, and in the other by palliative chemotherapy, as this patient had developed distant metastases as well.

Notably, the surgery followed by CCRT cohort demonstrated a statistically significant association with LF, as evidenced by 11 cases and a median duration of 7.9 months (range 2.1-26.6). For RF (3 cases, median 7.9 months, range 6.5-14.7) and DM (8 cases, median 6.9 months, range 3.9-7.9), no statistically significant associations were observed. Among patients in this cohort who developed LF, four were managed with chemotherapy alone, three were managed with re-RT, and one was managed with salvage surgery followed by adjuvant CCRT. Among the three patients in this cohort who developed RF, all three had previously undergone unilateral neck dissection. Two developed RR in the contralateral previously undissected neck at levels IV and VIII, while the other patient developed RR in the ipsilateral previously dissected neck at level I. One was managed by re-RT, one was managed by salvage surgery followed by adjuvant re-RT, and one was managed by salvage surgery followed by adjuvant CCRT. Finally, among patients who developed DM without LF or RF, six were found in the cohort treated with surgery followed by adjuvant RT, and five were found in the cohort treated with surgery followed by adjuvant CRT. Five of these patients’ DM were managed by RT, while three were managed by palliative chemotherapy.

### Predictors of survival

**Table 4** describes the multivariate analysis of prognostic factors for OS, DFS and DM. In the comparison of treatment cohorts, patients who underwent surgery followed by CCRT did not show a significant difference in OS compared to those who had surgery alone (HR=3.135, 95% CI 0.643-15.278, p=0.157). Similarly, the combination of surgery followed by adjuvant RT did not demonstrate a significant impact on OS compared to surgery alone (HR=1.819, 95% CI 0.400-8.273, p=0.438). The corresponding results for DFS were also not statistically significant.

**Table 4:** Multivariable analysis of prognostic factors for overall survival, distant metastases free survival and disease-free survival.

Variable	Overall survival		Disease-free survival		Distant metastases	
	HR (95% CI)	*p* value	HR (95% CI)	*p* value	HR (95% CI)	*p* value
pT stage category *pT3 + pT4 vs. pT1 +p T2*	1.136 (0.539-2.395)	0.736	1.428 (0.802-2.545)	0.226		
Tumor grade group *G2 + G3 vs. G1*	2.386 (0.994-5.727)	0.051			8.477 (1.127-63.776)	**0.037**
LVI *Present vs. Absent*	2.034 (1.001- 4.136)	**0.049**			3.380 (1.291-8.850)	**0.013**
Margins *Negative vs. Close*	0.905 (0.413-1.983)	0.803				
Margins *Positive vs. Close*	2.111 (0.863-5.166)	0.101				
Radiotherapy *Yes vs. No*	2.968 (0.683-12.899)	0.146				

pT, pathological tumor category; G, grade; LVI, lymphovascular invasion. Bold *p*-values denote statistical significance (P < 0.05).

Examining the impact of tumor stage (pT stage group), patients with T3 + T4 stages did not exhibit a significantly different OS compared to those with T1 + T2 stages (HR=1.136, 95% CI 0.539-2.395, p=0.736). The corresponding results for DFS were also not statistically significant. However, the tumor grade group G2 + G3 showed a trend towards worse OS compared to G1 (HR=2.386, 95% CI 0.994-5.727, p=0.051). This difference was more pronounced in DM, where the hazard ratio was 8.477 (95% CI 1.127-63.776, p=0.037).

The presence of LVI emerged as a significant prognostic factor regarding both OS (HR=2.034, 95% CI 1.001-4.136, p=0.049) and DM (HR=3.380, 95% CI 1.291-8.850, p=0.013). Margins status (negative vs. close, positive vs. close) did not significantly affect OS. However, the comparison of positive margins to close margins showed a trend towards worse OS for those with positive margins (HR=2.111, 95% CI 0.863-5.166, p=0.101). Similarly, the use of RT did not yield a statistically significant impact on OS, with a hazard ratio of 2.968 (95% CI 0.683-12.899, p=0.146).

## Discussion

This study from Jordan shows the oncologic outcomes of patients with OCSSC who were treated with curative surgery, surgery followed by RT, or surgery followed by CCRT at KHCC. This study demonstrated a 3‐year OS and DFS of 60.9% and 55.1%, respectively for the entire cohort. The presence of LVI and tumor grade group were both found to be significantly associated with DM, while only the presence of LVI was found to be associated with OS. The patients who received surgery followed by CCRT were significantly more likely to develop LF. Although treatment of OCSCC needs specialized care with advanced surgical and radiation oncology techniques, our outcomes from a developing country are consistent with the outcomes of treating OCSCC in developed countries.

116 cases of OCSCC were included in our study. 59.5% of them had T1-T2 disease, while 40.5% had T3-T4 disease. Of the former group, 64.8% required adjuvant RT, while 37.2% required adjuvant CCRT. These percentages are higher than those reported in a UK study (13), where only 19.1% of T-T2 cases required adjuvant RT and 2.6% required adjuvant CCRT. However, most of the reported cases in that study were N0, unlike ours. The most common subsites were the oral tongue (58.6%), followed by the buccal mucosa (14.7%), the floor of the mouth (9.5%) and the retromolar trigone (8.6%). These results are similar to those of two other studies in the UAE, in which the most common subsites were also the oral tongue and the buccal mucosa (14, 15). However, another study covering the epidemiology of oral cancer in Arab countries reported the most common sites to be the oral tongue and the lips (4). This is in contrast to our study, in which no squamous cell carcinomas of the lip were reported. This is due to the different classification of tumors at our institution, where lip cancers are classified as skin cancers, rather than cancers of the oral cavity anatomic subsite which was proven to be associated with tumor size and prognosis (16). One study in the UAE showed better prognosis for cancers of the tongue and buccal mucosa (14); meanwhile, another study found cancers of the oral tongue to be associated with worse OS, and higher rates of LF and DM (16).

Tobacco and alcohol consumption are widely recognized as primary risk factor for OCSSC, whereas in our region, the significance of alcohol is obscured by social and religious constraints (5). Notably, 93.5% of the patients in our study were smokers. This is similar to the results of other countries in the region, namely Egypt, Iraq and Libya, where more than half of patients afflicted with OCSSC were smokers (4). Smoking, both of cigarettes and hookah (water-pipe), is highly prevalent in Jordan – 57.4% of males and 12.7% of females are current smokers, amounting to 2.3 million smokers across the country, and leading to 3.2 thousand annual deaths attributable to smoking (17). Even more worrisome is the prevalence of smoking among the youth of Jordan. According to the World Health Organization’s (WHO) Global Youth Tobacco Survey (18), 23.2% of students aged 13-15 in Jordan are current tobacco smokers, with another 18.2% describing themselves as having never smoked, but are susceptible to tobacco use in the future. A case-control study conducted in Jordan found that hookah smoking especially was an independent risk factor associated with development of oral cancer at a younger age, and that hookah smokers were significantly younger when diagnosed with oral cancer (19).

Our study demonstrated a 3‐year OS of 60.9%. This is comparable to a Portuguese study, in which 3-year OS was 58.6% (20). A Chinese study done on OCSSC patients under the age of 45, however, demonstrated a 3-year OS rate of 77%; this is consistent with other published series showing a relatively better survival among younger patients (21). Multiple factors are known to influence OS, including notably the invasion of tumor cells into surrounding tissue, whether it be blood or lymphatic vessels. In our study, the presence of LVI was significantly associated with worse OS (HR = 2.034, *p* = 0.049). These findings align with outcomes from other studies that have shown LVI as an independent adverse prognostic factor, linked to worse OS (22, 23). Additionally, while pT stage, histologic grade and margin status were not found to be significantly associated with OS in this study, other studies conducted elsewhere have confirmed the significance of the association (20, 24–26). DFS at 3 years in our study was found to be 55.1%, similar to the forementioned Portuguese study in which 3-year DFS was 55.4% (20). Neither treatment cohort nor pT stage were found to be associated with DFS in our study. Meanwhile, other studies have previously found that advanced tumor stage, in addition to other factors such as PNI, node-positive disease and positive margin status, were associated with worse DFS (20, 21, 25, 26).

Regarding DM, both advanced tumor grade (HR = 8.477, *p* = 0.037) and the presence of LVI (HR =3.38, *p =* 0.013) were found to be significantly associated with higher potential for DM. The presence of LVI in particular indicates that tumor cells have entered the vascular compartment, heralding the potential for metastases (27). These results are consistent with those of other studies, which found risk factors for the development of DM to include advanced tumor grade and LVI, in addition to other factors such as tumor thickness, pENE, and lymph node metastases to levels IV and V (28–30).

The primary contributors to mortality related to OSCC are local and regional recurrences, with the 5-year survival rate declining from 92% in patients without recurrence to 30% in those experiencing recurrence (31, 32). In our study, 25 patients (21.5%) developed LF, 10 patients (8.6%) developed RF and 18 patients (15.5%) developed DM. In comparison, studies from India, Qatar and China demonstrated LF rates of 2.72%, 11.7%, and 9.7% respectively (26, 32, 33). Additionally, in the Indian and Chinese studies, and in a study from Taiwan, RF rates were found to be 4.7%, 12.5%, and 32.7% respectively (26, 31, 32). Hence, while RF rates at our center appear to be similar to those found in other countries, LF rates appear to be higher. This may be attributable to the high prevalence of poor pathologic characteristics found in our patients, such moderate to poor differentiation of tumors (73.7%), close or positive margins (61.8%), and T3-T4 stage tumors (47%). Additionally, in our study, the cohort of patients treated by surgery followed by CCRT was significantly more likely to develop LF. According to available literature, the 5-year survival rates for these patients with locally advanced disease range from 11% to 64%. Relapse occurs in about one-third of patients, with locoregional recurrence being the most frequent pattern of failure (34). This may be attributable to the poor tumor characteristics seen in this high-risk group, such as positive postsurgical margins and ENE, which are known to be risk factors for reduced OS and LF (35).

Our study has a number of limitations. Firstly, the study’s retrospective nature may introduce selection bias. Additionally, as this is a single-institution study, the generalizability of findings to a broader population may be somewhat limited. Thirdly, patients with lip OCSSC were not included as an OCSCC in this study, as they are classified in our institution as skin cancers. Furthermore, we did not collect treatment related toxicity in this study.

## Conclusions

We present the clinical outcomes of a cohort of patients with OCSCC treated with surgical resection alone, surgery followed by postoperative RT, or surgery followed by CCRT in Jordan. Those who received adjuvant CCRT were more likely to have LF, and LVI was associated with worse OS and DM. These outcomes are concordant with the known literature about the treatment of OCSCC.

## Data Availability

The raw data supporting the conclusions of this article will be made available by the authors, without undue reservation.
